# Complexes of Pro-Apoptotic siRNAs and Carbosilane Dendrimers: Formation and Effect on Cancer Cells

**DOI:** 10.3390/pharmaceutics11010025

**Published:** 2019-01-10

**Authors:** Olga A. Krasheninina, Evgeny K. Apartsin, Elena Fuentes, Aleksandra Szulc, Maksim Ionov, Alya G. Venyaminova, Dzmitry Shcharbin, F. Javier de la Mata, Maria Bryszewska, Rafael Gόmez

**Affiliations:** 1 Institute of Chemical Biology and Fundamental Medicine SB RAS, 630090 Novosibirsk, Russia; okrasheninina@gmail.com (O.A.K.); ven@niboch.nsc.ru (A.G.V.); 2 Departamento de Química Orgánica y Química Inorgánica, UAH-IQAR, Universidad de Alcalá, 28805 Alcalá de Henares, Spain; elena.fuentes.paniagua@gmail.com (E.F.); javier.delamata@uah.es (F.J.d.l.M.); 3 Networking Research Center on Bioengineering, Biomaterials and Nanomedicine (CIBER-BBN), Madrid, Spain; 4 Department of General Biophysics, University of Lodz, 90-236 Lodz, Poland; aleksandra_szulc@interia.pl (A.S.); maksim.ionov@biol.uni.lodz.pl (M.I.); maria.bryszewska@biol.uni.lodz.pl (M.B.); 5 Institute of Biophysics and Cell Engineering of NASB, 220072 Minsk, Belarus; d.shcharbin@gmail.com; 6 Instituto Ramón y Cajal de Investigación Sanitaria, IRYCIS, Madrid, Spain

**Keywords:** carbosilane, dendrimers, anticancer siRNA, nucleic acids, dendriplexes, complexation, transfection, cytotoxicity, biophysical assays

## Abstract

This paper examines the complexation of anti-cancer small interfering RNAs (siRNAs) by cationic carbosilane dendrimers, and the interaction of the formed complexes with HeLa and HL-60 cancer cells. Stepwise formation of the complexes accompanied by the evolution of their properties has been observed through the increase of the charge ratio (dendrimer/siRNA). The complexes decrease the viability of both “easy-to-transfect” cells (HeLa) and “hard-to transfect” ones (HL-60), indicating a high potential of the cationic carbosilane dendrimers for siRNA delivery into tumor cells.

## 1. Introduction

Therapeutic nucleic acids hold great potential for anti-tumor therapy. However, the natural barriers of a cell form considerable obstacles against the efficient transport of nucleic acids (NA) into cytosol or target compartments [1,2,3,4]. Nowadays, a wide number of methods for the transfer of the nucleic acid material (such as plasmid DNA or messenger RNA (mRNA)) and regulatory oligonucleotides (such as small interfering RNAs (siRNAs), microRNAs, antisense oligonucleotides, guide RNAs for CRISPR/Cas9 system, etc.) into a cell has been developed [5,6,7]. As carriers for NA, various chemically designed systems from small hydrophobic and positively charged moieties to soft and hard nanoparticles-based constructions have been tested [8,9]. Among the NA-delivery systems explored so far, supramolecular associates incorporating nucleic acid cargo are relatively simple and synthetically accessible systems, which are characterized by controllable composition, insufficient cytotoxicity, and high transfection efficiency. In addition, these associates may protect NA from detrimental intracellular nucleases, and provide the release of the agents in a controlled manner. Dendritic compounds bearing cationic groups on their periphery often serve as building blocks for supramolecular NA-delivery systems [10,11,12]. Dendritic macromolecules are highly symmetrical tunable hyperbranched polymers with a well-defined core, branches arranged in a repetitive manner, and variable peripheries. Cationic dendritic systems and NA assemble into complexes, mainly by the means of electrostatic interactions, with their sizes and properties depending on a dendrimer/NA charge ratio. These complexes, called dendriplexes, may easily penetrate across the cell membrane by endocytosis, and release the NA cargo inside a cell.

Among many types of dendrimers described so far, cationic carbosilane dendritic systems gain considerable attention as potential NA-based drug transporters, due to their good biocompatibility, chemical stability, highly controlled size, inert inner branches and high charge density on the surface. To date, their utility has been demonstrated for the efficient delivery of model antisense oligonucleotide-targeted mRNA of the Renilla luciferase gene [13], pro-apoptotic siRNAs [14,15] and siRNA blocking the expression of transcription factor HIF-1α [16] in vitro and in vivo. Moreover, the comprehensive study [17] reports a successful transport of siRNA inhibiting HIV-1 infection to HIV-infected human primary astrocytes, and, more interestingly, crossing the blood-brain barrier in mice. More recently, sophisticated nanoconstructions based on cationic carbosilane dendrons, namely noncovalently dendronized carbon nanotubes [18] and spherical dendritic micelles formed by amphiphilic dendrons [19] have been designed as potential nanocarriers for therapeutic NA.

Here we report on a detailed physico-chemical study of the formation and stability of dendriplexes formed by cationic carbosilane dendrimers and pro-apoptotic siRNAs. Also, we evaluated the cell cytotoxicity of the dendriplexes towards cancer cell lines of different permeability, namely, the suspension human acute promyelocytic leukemia cell line (HL-60) and the adherent human cervical cancer cell line (HeLa).

## 2. Materials and Methods

**Figure 1:**
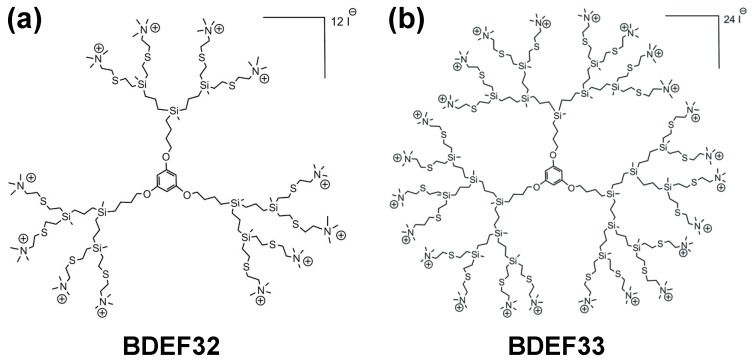
Molecular structures of the cationic carbosilane dendrimers of the (**a**) second (BDEF32) and (**b**) third (BDEF33) generation used in the study.

Cationic carbosilane dendrimers with peripheral quaternary ammonium groups were prepared and characterized according to the reported method [20]. The molecular structures of the dendrimers are presented in Figure 1.

The synthesis of oligoribonucleotides was carried out on an automatic ASM-800 DNA/RNA synthesizer (Biosset, Novosibirsk, Russia) at the 0.4 µmol scale, with the use of 2′-*O*-*tert*-butyldimethylsilyl-protected RNA phosphoramidites (5-(ethylthio)-1H-tetrazole as an activator; the coupling time was 5 min) and with automated procedures being optimized for the synthesizer. Cleavage from a solid support, and the removal of protecting groups of oligoribonucleotides were performed under the conditions described in [21]. Unprotected oligonucleotides were purified by denaturing polyacrylamide gel electrophoresis (PAGE, 15%) and desalted, and then the pure oligonucleotides were precipitated as Na^+^-salts. The identities of the oligoribonucleotides were verified by MALDI-TOF mass spectrometry (MS) analysis (Table S1). The MS spectra of the oligoribonucleotides were recorded on a REFLEX III spectrometer (Bruker Daltonics, Billerica, MA, USA) with the use of 3-hydroxypicolinic acid as a matrix. The lyophilized sense and antisense strands (see Table S1) of the siRNAs were dissolved in a buffer containing 137 mM NaCl, 2.7 mM KCl, 10 mM phosphate buffer, pH 7.4, and the final concentration of the siRNA was 50 μM. The solution was heated at 90 °C for 2 min, then slowly cooled to the room temperature for 1 h.

Dendriplexes were formed by combining negatively charged siRNA (see concentrations below) and positively charged carbosilane dendrimers in a RNase free 1× phosphate-buffered saline (PBS) buffer (10 mM phosphate buffer, pH 7.4, 137 mM NaCl, 2.7 mM KCl) with the following incubation for 10 min at 25 °C. The dendrimer:siRNA ratio was calculated as:

$$CR=\frac{N_{+}C_{D}}{N_{-}C_{siRNA}}=\frac{N_{+}}{N_{-}}MR$$

where CR is the charge ratio; $MR=\frac{C_{D}}{C_{siRNA}}$ is the molar ratio; $N_{+}$ is the number of cations per dendrimer molecule (12 for BDEF32 or 24 for BDEF33); $N_{-}$ is the number of anions per siRNA molecule (40 for Bcl-2, Bcl-xL, Mcl-1, or 42 for Scr).

### 2.1. Gel Retardation Assay

The ability of the cationic carbosilane dendrimers to form complexes with siRNAs was studied by gel electrophoresis in 1% agarose gel. Dendriplexes were prepared by mixing siRNA (40 pmol per band), ethidium bromide (EB) (0.4 µM, ~1 EB molecule per 2 bp of siRNA) and dendrimers (at increasing concentrations, depending on the charge ratios), dissolved in 1×PBS. After 30 min incubation at 25 °C, electrophoresis was run in 1% agarose gel at 80 V (Mini-Sub^®^ Cell GT, Bio-Rad, Hercules, CA, USA) in 1×TBE buffer, pH 8.4, and the bands were visualized under UV using a gel documentation system (Helicon, Moscow, Russia).

### 2.2. Ethidium Bromide Intercalation Assay

Samples containing ethidium bromide (EB) and siRNAs at final concentrations of 3 and 0.3 µM, respectively (~1 EB molecule per 2 bp of siRNA), were prepared in 1×PBS (10 mM phosphate buffer, pH 7.4, 137 mM NaCl, 2.7 mM KCl) and incubated at 25 °C for 10 min, then the increasing amounts (final concentrations were in the range from 0 to 9 µM) of dendrimers were added to the samples. The fluorescence spectra of EB were recorded in the region of 500–800 nm (excitation wavelength was 480 nm) using PerkinElmer LS 50-B spectrofluorometer (PerkinElmer, Waltham, MA, USA). The excitation and emission slit widths were set at 10 and 15 nm, respectively. The data are expressed as mean values ± SD (at 595 nm) of five independent experiments for both generations of dendrimers.

### 2.3. Zeta Potential Measurements

The zeta potential values of dendriplexes were measured by laser Doppler electrophoresis (electrophoretic light scattering). All measurements were performed using the Zetasizer Nano-ZS (Malvern Instruments Ltd., Malvern, UK). Samples of 0.5 µM siRNAs were prepared in 10 mM phosphate buffer, pH 7.4, then the increasing amounts of dendrimers (final concentrations were in the a range from 0 to 9 µM) were added to the samples with the following incubation for 10 min at 25 °C. The saturated dendriplexes were treated with heparin. The zeta potentials of the complexes were determined from electrophoretic mobility using the Smoluchowski approximation. The data are expressed as the mean values ± SD of five independent experiments for both generations of dendrimers.

### 2.4. Circular Dichroism–Spectroscopy

The circular dichroism (CD)-spectra of the dendriplexes were recorded using a JASCO J-815 circular dichroism spectropolarimeter (JASCO, Tokyo, Japan). Samples containing 2 µM siRNA were prepared in 1×PBS (10 mM phosphate buffer, pH 7.4, 137 mM NaCl, 2.7 mM KCl), and then increasing amounts of dendrimers (final concentrations were ranged from 0 to 15 µM) were added to the samples. Then, the saturated dendriplexes were treated by heparin.

### 2.5. AFM Experiments

Dendriplexes were obtained by mixing of solutions of siRNA (1 µM) and dendrimer at charge ratio 1:5. An aliquot of dendriplex solution was deposited onto a mica slide for 1–2 min. The slide was then washed 3 times with deionized H_2_O and dried on air. Scanning was performed in tapping mode using Multimode 8 atomic force microscope (Bruker AXS, Karlsruhe, Germany) with NSG10_DLC cantilevers with tip curvature radii of 1–3 nm (NT-MDT, Moscow, Russia), at scanning rate of 3 Hz. Images were processed using Gwyddion 2.36 software (Czech Metrology Institute, Brno, Czech Republic).

### 2.6. Cell Culture and Transfection

The adherent human cervical cancer cell line (HeLa) was purchased in Banca Biologica e Cell Factory (Genova, Italy), and a suspension human acute promyelocytic leukemia cell line (HL-60) was purchased in ATCC (Manassas, VI, USA). HeLa cells were cultured in Dulbecco’s Modified Eagle Medium (DMEM, Gibco, Poland), and HL-60 cells were cultured in Roswell Park Memorial Institute medium (RPMI-1640, Sigma Aldrich, Poznan, Poland) in a humidified incubator containing a mixture of air and 5% of CO_2_ at 37 °C (Brunswick, Lake Forest, IL, USA). DMEM and RPMI-1640 were supplemented with 10% fetal bovine serum (FBS), 100 U/mL penicillin and 100 µg/mL streptomycin (Sigma Aldrich, Poznan, Poland). The viability of cells was evaluated by counting the cells after dyeing with 0.2% Trypan blue.

For the cell viability experiments, cells (2 × 10^4^ cells in 100 µL per well) were cultured in 10% FBS containing DMEM or RPMI-1640 for 24 h on 96-well microplates. Complexes of siRNAs (final concentrations in wells were 0, 50, 100, and 250 nM) and dendrimers at a charge ratio of 1:10 were prepared in 1×PBS and incubated for 15 min at 25 °C. After treatment of the cells by siRNAs, dendrimers and dendriplexes, the cells were incubated for 72 h in a humidified incubator containing a mixture of air and 5% CO_2_ at 37 °C.

### 2.7. In Vitro Cytotoxicity Assays

After 72 h treatment of the cells by dendrimers or dendriplexes in the corresponding serum-containing media (see above), the media were refreshed. The influences of siRNA, dendrimers, and dendriplexes on the cell viability was determined using the 3-(4,5-dimethylthiazol-2-yl)-2,5-diphenyltetrazolium bromide (MTT) cell viability assay for the HeLa cell line [22], and the spectrofluorimetric resazurin assay (SRA) or Alamar Blue assay for the HL-60 cell line [23,24].

In the MTT assay, yellow MTT (3-(4,5-dimethylthiazol-2-yl)-2,5-diphenyltetrazolium bromide) is reduced by the cellular reductases of viable cells to a dark blue formazan [22,25]. MTT was added to each well at final concentration of 0.5 mg/mL and the cells were incubated for 3 h in 5% CO_2_ humidified incubator at 37 °C. After 3 h of incubation, MTT solution was removed, and DMSO was added to dissolve the formazan crystals [22,25]. The absorption of the samples was measured at the reference wavelength of 630 nm and a test wavelength of 570 nm, using a microplate spectrophotometer (BioTek, Burlington, VT, USA).

In the Alamar Blue assay, blue non-fluorescent resazurin is reduced to pink fluorescent resorufin, which is a metabolic response of living cells. This resazurin conversion determines the cell viability [26,27]. After incubation, 20 μL of resazurin solution (1 mg/mL in PBS) was added to each well, and the cells were incubated for 2 h at 37 °C in the dark. Then, resorufin fluorescence was read at λ_ex_ = 530 nm and λ_em_ = 590 nm by using a fluorescence microplate reader (Fluoroskan, Thermo Fisher Scientific Inc. Waltham, MA, USA). The cell viability was presented as a percentage of the fluorescence obtained for untreated control cells treated by 1×PBS.

### 2.8. Statistics

The Shapiro–Wilk test was used to check the normality of distribution. The results are presented as a mean ± SD (standard deviation), *n* = 6. The data were analyzed by a paired Student *t*-test.

## 3. Results and Discussion

### 3.1. siRNAs

A prospective method of nucleic acid-based anti-cancer treatment is the use of siRNAs to induce the apoptosis in target cells. The therapeutic effect is achieved by down-regulating the expression of the genes that are responsible for the maintenance of cell proliferation. From this point of view, a promising target is the Bcl-2 family of apoptosis-regulating genes [28]. This family, the key regulator of the mitochondrial apoptosis pathway, consists of both pro-apoptotic and anti-apoptotic genes. Whereas the expression of these genes is normally balanced to keep a cell proliferating, putting this equilibrium out of balance by silencing the anti-apoptotic genes leads to the activation of the Bax-mediated programmed cell death [29].

As therapeutic oligonucleotides, siRNAs targeted to the mRNAs of three anti-apoptotic genes of the Bcl-2 family (Bcl-2, Bcl-xL, Mcl-1) were chosen from previously published works [30,31]. As a scrambled siRNA (Scr), we chose a complementary pair of oligoribonucleotides that had no exact matching with the human genome [31]. The sequences of the oligoribonucleotides used are given in Table S1. The activity of these siRNAs and their cocktails (equimolar siRNA mixtures) against cancer cells upon dendrimer-mediated delivery has been demonstrated in other works [15,32].

### 3.2. Cationic Carbosilane Dendrimers

The dendrimers used in the study (BDEF32 and BDEF33, Figure 1) have a rigid 1,3,5-trihydroxybenzene core, which is close to surface of the dendrimers and accessible to the solvent; this forces the branches of the dendrimers to spread away from the core. For this reason, the dendrimers are less susceptible to steric hindrance during the synthesis of the branches (i.e., when the dendritic generation increases), and this makes them easy synthetically accessible, and, in addition, they possess higher chemical stability and solubility when compared to carbosilane dendrimers that are derived from a Si atom core [20].

The ammonium-terminated dendrimers G*_n_*O_3_[SNMe_3_I]*_m_* of the first (*n* = 1, *m* = 6), and second (*n* = 2, *m* = 12 (BDEF32)) generations (where G*_n_* indicates the generation, O_3_ is a core derived from 1,3,5-trihydroxybenzene, and [SNMe_3_I]*_m_* describes the peripheral function and its number (m)) have already been studied with respect to their antibacterial properties against Gram-positive (*Staphylococcus aureus* CECT 240) and Gram-negative *(Escherichia coli* CECT 515) bacterial strains [20]. These dendrimers were shown to be effective antibiotics against both Gram-positive and Gram-negative model strains, while the reference, the well-known antibiotic penicillin V potassium salt is active only against the Gram-positive *S. aureus* CECT 240 strain.

The study [17] demonstrates the successful use of the second-generation dendrimer G_2_O_3_[SNMe_3_I]_12_ (BDEF32) and its fluorescein isothiocyanate (FITC)-labeled analogue G_2_O_3_[SNMe_3_I]_11_-FITC, for the delivery of the specific anti-HIV-1 siRNA Nef to interfere with HIV-infectivity in human primary astrocytes. The work indicates that this type of transporters is a promising alternative to achieving very high transfection levels in “hard-to-transfect” astrocytes without causing cytotoxicity. The biodistribution of the dendriplex formed by the siRNA and the dendrimer loaded with FITC was studied in vivo in BALB/c mice. The dendrimer efficiently transferred the siRNA to mouse brains and it crossed the bloodαbrain barrier, showing its great potential as a drug transporter for the therapy of neurological disorders. Motivated by these inspiring results we have chosen this family of cationic dendrimers for the binding and transport of pro-apoptotic siRNAs to “easy-to-transfect” (HeLa) and “hard-to transfect” (HL-60) tumor cell lines. The cationic carbosilane dendrimers of the second and the third generations G*_n_*O_3_[SNMe_3_I]*_m_* (*n* = 2, *m* = 12 (BDEF32); *n* = 3, *m* = 24 (BDEF33)) were prepared according to the convenient procedure described in [20]. In brief, the functional groups on surface of the dendrimers were introduced by a “thiol-ene click chemistry” reaction taking place between spherical vinyl-functionalized dendrimers, and 2-(dimethylamino)ethanethiol hydrochloride, which is fast, easy initiated, chemoselective, and gives high yields. Then, neutralization and subsequent quaternization by the excess of iodomethane led to the ammonium-terminated dendrimers BDEF32 and BDEF33 (Figure 1). Structural characterization of the dendrimers has been carried out by using ^1^H- and ^13^C-NMR spectroscopy and mass spectrometry.

### 3.3. Formation of Dendriplexes

Cationic carbosilane dendrimers can bind siRNAs, mainly by the means of electrostatic interactions. The properties of the dendriplexes formed depend on their composition, i.e., on the charge ratio of the components taken. To study the ability of the dendrimers BDEF32 and BDEF33 to bind siRNAs, and the properties of the formed dendriplexes, and to find a ratio that could be considered optimal for the further cell experiments, the profiles of siRNA binding by cationic dendrimers were obtained by the means of several physico-chemical methods. The use of independent indirect methods to study siRNA binding is required, since each of these methods provides information on only on an exact property of the dendriplexes (charge, size, structure, etc.) [33]. Thus, it is possible to follow all the stages of the dendriplex formation: from initial binding through to structural changes in the RNA duplex, to the rearrangement of the complexes to yield particles that are as charged as possible.

**Figure 2:**
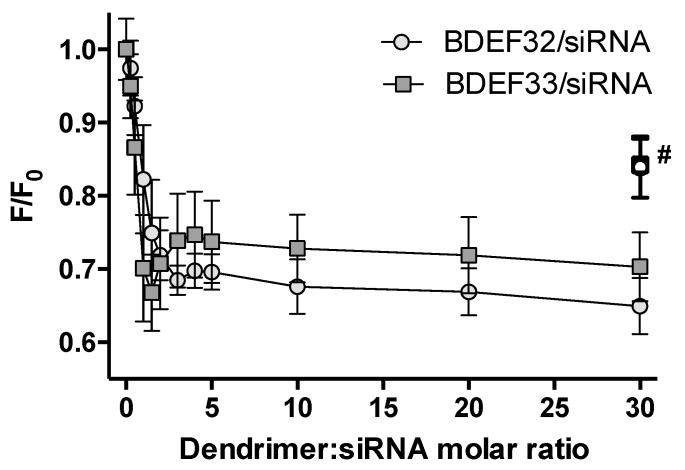
Change of the normalized fluorescence intensity (at 595 nm) of ethidium bromide upon titration of small interfering RNA (siRNA) (0.3 µM siRNA Bcl-xL, 3 µM ethidium bromide (EB)) by carbosilane dendrimers (# marks samples after heparin treatment). Conditions: 1×phosphate-buffered saline (PBS) (10 mM phosphate buffer, pH 7.4, 137 mM NaCl, 2.7 mM KCl), 25 °C.

The early stage of the siRNA binding to the dendrimers was studied by means of the ethidium bromide intercalation assay (EBIA). The method is based on the intercalation of the fluorescent dye EB into a double strand siRNA (a binding site of 2–4 base pairs). These interactions result in a significant increase of EB fluorescence intensity, and they cause a blue shift in EB fluorescence emission maximum. Strong electrostatic interactions between cationic dendrimers and siRNAs lead to a higher affinity of the dendrimers to siRNAs. The following structural distortions of siRNA causes the displacement of intercalated EB from the duplex and subsequent quenching of EB fluorescence. Thus, EBIA is a sensitive method for monitoring even subtle changes in double-stranded siRNA structure [33]. We observed a fast decrease of EB fluorescence intensity upon titration of EB–siRNAs complexes by the dendrimers BDEF32 and BDEF33 up to about equivalent charge ratios (~1:1, for both dendrimers and regardless of the siRNA structure) (see Figure 2 and Figure S1), reaching a plateau at ~3-fold molar excess of the dendrimers.

The reversible nature of the binding was confirmed by recovery of EB fluorescence intensity upon the treatment of the saturated dendriplexes with heparin, a natural polyanion [34]. When the dendriplexes were treated with heparin, the siRNA was released.

**Figure 3:**
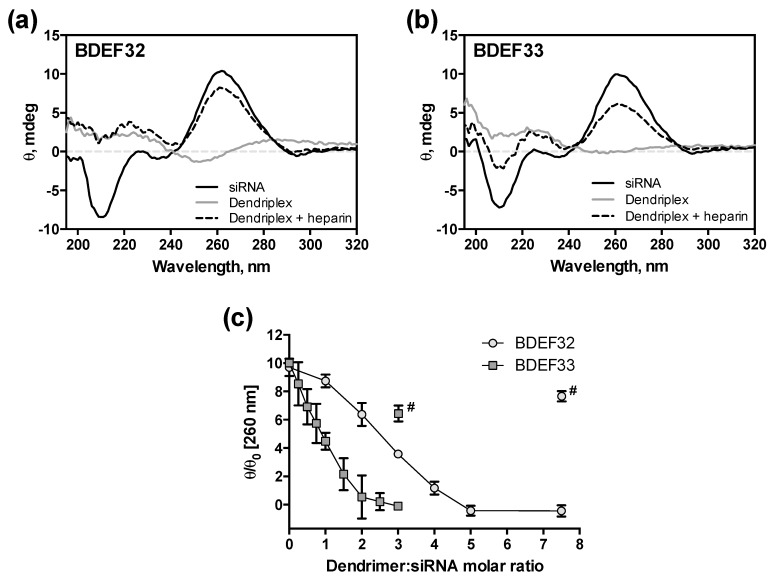
Circular dichroism (CD) spectra of siRNA Bcl-xL (2 µM) in the presence of the carbosilane dendrimers of (**a**) the second ([BDEF32] = 15 µM) and (**b**) the third generations ([BDEF33] = 6 µM); (**c**) changes of ellipticity (at 260 nm) of the dendriplexes formed by siRNA Bcl-xL and dendrimers BDEF32 and BDEF33 (# marks samples after heparin treatment). Conditions: 1×PBS (10 mM phosphate buffer, pH 7.4, 137 mM NaCl, 2.7 mM KCl), 25 °C.

Further evolution of the double-stranded RNA structure upon the binding with dendrimers was studied by CD spectroscopy. The CD spectra of the naked siRNAs indicate the typical curve of an A-form helix geometry, with a strong positive band at ~260–265 nm, and a negative band at ~210 nm (Figure 3a,b). Since the binding of siRNAs to dendrimers causes a disturbance in their structures, titration of the siRNAs by the dendrimers was accompanied by a significant decreases of intensities of the characteristic bands to completely smooth out the spectra (Figure S2). The double-stranded structure of the siRNA appeared to be completely distorted at a siRNA:dendrimer molar ratio 1:2.5 (G3) or 1:5 (G2) (Figure 3c). Both the values of molar excess corresponded to the 1.5-fold excess of cations. Moreover, after addition of the dendrimers to siRNAs, we observed red-shifts in the CD spectra maximum from ~260 nm to ~280 nm, and in the corresponding minimum from ~210 nm to ~215 nm. The addition of heparin to the saturated dendriplexes reduced the initial shape of the siRNAs CD spectra, indicating the release of native siRNAs from the complexes as a duplex (Figure 3c). This finding is important, since the duplex is the biologically active siRNA form. It also suggests that the base pairing of the duplex strands is not fully disrupted when it is bound to dendrimer.

**Figure 4:**
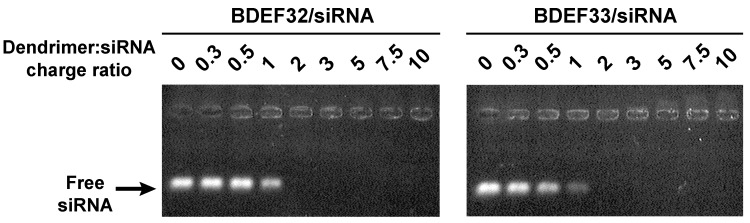
1% Agarose gel electrophorgrams of Mcl-1 siRNA/carbosilane dendrimers of the second (BDEF32) and the third (BDEF33) generations, complexed at the corresponding charge ratio. Samples containing 40 pmol siRNA per line, 0.4 µM EB, 1 EB per 2 bp of siRNA, and dendrimer were prepared in 1×PBS (10 mM phosphate buffer, pH 7.4, 137 mM NaCl, 2.7 mM KCl). Gels were visualized upon transillumination at 365 nm.

In addition, the complexation was studied by the means of an agarose gel retardation assay. This method is based on the mobility of the charged macromolecules (or complexes of molecules) in an electric field through a porous lattice formed by agarose gel, with the retardation of the molecules depending on their charge and mass. Herein, it allowed us to observe the non-complexed siRNA in samples. The electrophoresis data show that the siRNA was fully complexed by cationic dendrimers, being in 2-fold charge excess, which corresponded to a 6.7-fold molar excess (BDEF32) or a 3.3-fold molar excess (BDEF33), respectively (Figure 4). The complexes formed were cationic dendriplexes that were retained at the start line, or that migrated towards the cathode (Figure 4, right).

**Figure 5:**
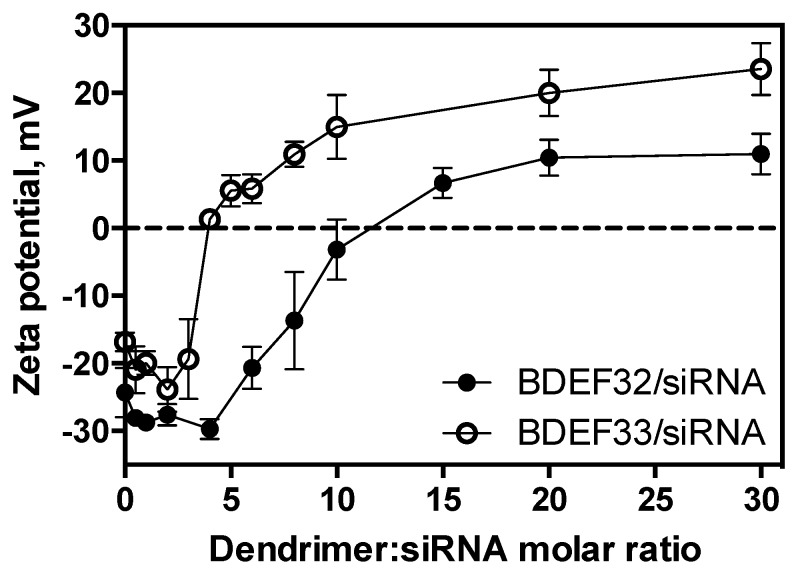
Zeta potential profiles of the dendriplexes (0.5 µM siRNA Mcl-1) as a function of the concentration of carbosilane dendrimers of the second (BDEF32) and third (BDEF33) generation. Conditions: 10 mM phosphate buffer, pH 7.4, 25 °C.

In the experiments, we observed the initial moment when stabile supramolecular associates were formed. The increase of the dendrimer/siRNA ratio led to the further evolution of the dendriplex structure, with the reorganization of the particles and the increase of their size and surface charge. The changes could be monitored by the means of electrophoretic light scattering. The technique was based on the scattering of the laser light by nanoscale objects moving in the electric field. The value of the potential on the border of the solvate shell (zeta potential) calculated from the scattering data gives the idea of the surface charge of nanoparticles. The profiles had a sigmoidal shape; negative charges of siRNAs were compensated at the 12-fold excess of the G2 dendrimer, and at the 4-fold excess of the G3 one. A further increase of the dendrimer concentration led to a charge saturation at the 20-fold (G2) or 10-fold (G3) molar excesses of dendrimers (Figure 5 and Figure S3).

The results obtained from the biophysical assays described above are summarized in the Table 1. The molar ratio (MR) values represent the molar excess of dendrimers to siRNAs. Herein, since dendrimers bearing pH-independent charged groups have been used, it is also possible to operate charge ratio (CR) values, i.e., the excess of cationic groups in complexes. The comparison of the CR values seems to be more representative if dendrimers of different generation are taken, for it reveals the role of the number of charges per dendrimer molecule in the complexation. The dendritic effect is also better seen if analyzed in the terms of CR.

In the complexation profiles, two points are of special interest: (1) half-effect point (MR_50_, CR_50_) corresponding to the charge or molar ratio of the half-binding; and (2) the saturation point (MR_sat_, CR_sat_) corresponding to the charge or molar ratio where the measured parameter is no longer changed Whereas the former point is convenient for observing dendritic effects, the latter represents the properties of dendriplexes at the end of the complexation.

Analyzing the data from the assays studying the dendriplexes’ formation, one could find an interesting regularity in the results obtained: the binding and saturation values that were obtained by CD spectroscopy, EBIA, and agarose gel electrophoresis were quite close one to another (CR_50_ was estimated by 1, a small positive dendritic effect is observed; CR_sat_ is ~1.5–2). In contrast, the zeta potential values continue to evolve even after the saturation achieved, as shown by other assays (at a CR_50_ value of 4 (for G2) or 2.4 (for G3), a strong positive dendritic effect is observed; CR_sat_ is 6 for both generations).

The observed differences suggest that two processes occur upon the exposition of siRNAs to cationic dendrimers. The first one is the binding of siRNA to dendrimers. It is accompanied by the distortion of the NA duplex structure (refer to the CR_50_ values in the Table 1). After that, during the increasing of the dendrimer concentration (i.e., molar and charge excess) dendriplex particles appear to be rearranged due to the reversibility of the dendriplexes’ structures [35]; siRNA molecules are surrounded by more dendrimer molecules than before. This leads to an increase of the surface charge of the dendriplex particles.

Previously, the appearance of plural CR_50_/MR_50_ values upon the siRNA binding by cationic dendrimers was observed [36,37], with minor differences having been found upon changing the dendrimers’ architecture (refer, for example, to the comparison of the siRNA binding by polyamidoamine, phosphorus and carbosilane dendrimers [14,15]). However, to the best of our knowledge, the stepwise dendriplex formation has never been deduced from these findings. The work [35] analyzes rearrangements of dendriplexes in the context of the dendriplexes’ lability towards spontaneous decomposition into smaller complexes.

It should be noted that the stepwise formation of dendriplexes have never been observed upon the binding of NAs to dendrimer-based nanoparticles. The surface charge of dendriplex nanoparticles is stabilized at relatively low CR/MR values, corresponding to the full NA binding. However, similar behaviors has been observed for amphiphilic PAMAM dendrons [38,39] and dendrimers [40], amphiphilic carbosilane dendrons [19], and dendron-decorated carbon nanotubes [18].

**Figure 6:**
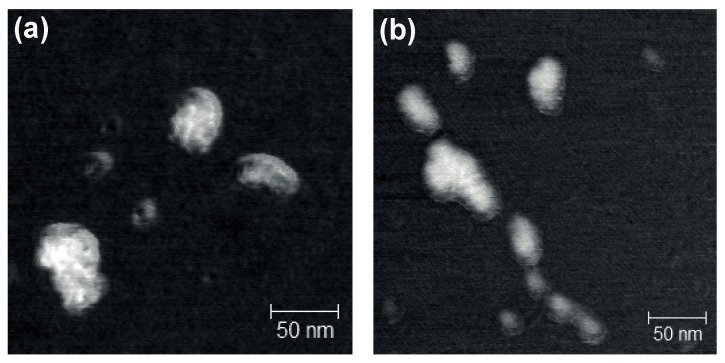
Atomic force microscopy images of the dendriplexes of carbosilane dendrimer (**a**) BDEF32 and (**b**) BDEF33 with siRNA Bcl-2. The charge ratio is 5.

One can speculate whether the dendriplex composition (i.e., charge/molar ratio) is optimal for biological experiments. Indeed, the initial intention is to keep the dendrimer content in samples as low as possible, to avoid unwanted side effects, for instance, increased cytotoxicity. However, the use of higher dendrimer excess likely leads to the decrease of the particle size [34,41,42], with the size evolution profiles correlating with those of zeta potential. This factor is important for the efficient cell internalization of dendriplexes, since it occurs by means of clathrin- or caveolin-mediated endocytosis [43,44], where the size of nanoparticles to be internalized is limited. Summarizing, the use of CR_sat_/MR_sat_ obtained from the zeta potential profiles for the preparation of dendriplexes is likely preferential for the biological experiments (at least, in vitro). The AFM images of the dendriplexes prepared at the CR value of 5, which is close to the saturation region, are given in the Figure 6. The size of the dendriplexes varies from 35 to 75 nm.

### 3.4. Effect of Dendrimers and Dendriplexes on Cancer Cells

As target cell lines, HeLa (cervical carcinoma cells) and HL60 (human myeloid leukemia cells) were chosen to study the influence of the cells’ characteristics on the transfection efficiency. The lines under study represented two major types of the human cancer cell lines: adherent (HeLa) and suspension (HL60) lines. Due to the peculiarities of the cell membrane composition, HL60 cells are known as hard-to-transfect [45], whereas HeLa cells are normally considered as easy-to-transfect cell lines.

**Figure 7:**
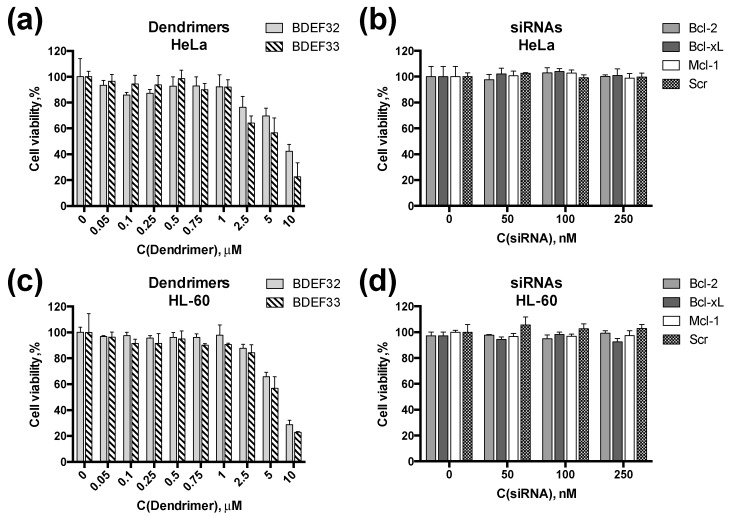
Cytotoxicities of dendrimers and siRNAs. Viability of HeLa cells treated with (**a**) dendrimers BDEF32 and BDEF33; or (**b**) pro-apoptotic siRNAs, as measured by the MTT assay. Viability of HL-60 cells treated with (**c**) dendrimers BDEF32 and BDEF33; or (**d**) pro-apoptotic siRNAs measured by spectrofluorimetric resazurin assay (SRA). The viability of the cells was evaluated (% relative to the negative control) after 72 h incubation with dendrimers and siRNAs.

Both types of dendrimers exhibited dose-response cytotoxicities towards HeLa and HL60 cells. The cytotoxicity profiles looked similar for both cell lines, thus suggesting that leukemia cells (HL60) are principally permeable by dendrimers (Figure 7a,c). As expected, siRNAs had no effect on the cell viability (Figure 7b,d).

Complexes of carbosilane dendrimers and siRNAs were shown to penetrate efficiently across the cell membrane [15]. Herein, we assessed the effects of three anti-cancer siRNAs, Bcl-2, Bcl-xL, and Mcl-1, on the viability of HeLa and HL60 cancer cells. All of these siRNAs inhibit the synthesis of anti-apoptotic proteins of the Bcl-2 family, thus directing target cells to the Bax-mediated apoptosis. As a control, scramble siRNA, which was claimed not to have any target sequence in the human genome [31] was taken.

Upon treating target cells with anti-cancer dendriplexes, several consecutive processes take place: dendriplexes’ endocytosis, endosomal escape, siRNA release and functioning, apoptosis induction [32]. A deficiency at any step is supposed to result in a sharp decrease of the overall effect, i.e., cell viability. In view of this, measuring cell viability can be a convenient technique for a fast screening of the dendriplexes’ anti-cancer activity.

**Figure 8:**
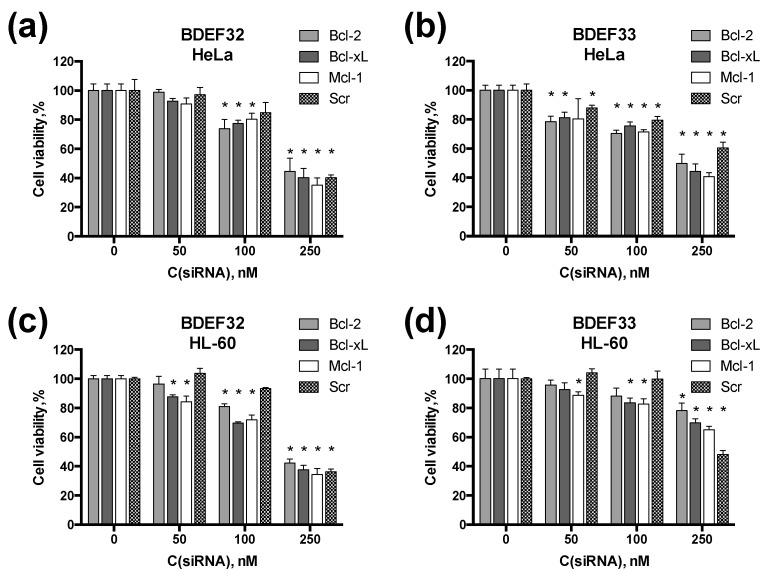
Cytotoxicity of dendriplexes. Viability of HeLa cells treated with dendriplexes formed by (**a**) BDEF32 and (**b**) BDEF33 and pro-apoptotic siRNAs, as measured by the MTT assay. Viability of HL-60 cells treated with dendriplexes formed by (**c**) BDEF32 and (**d**) BDEF33 and pro-apoptotic siRNAs, as measured by spectrofluorimetric resazurin assay (SRA). Molar ratio of [dendrimer]/[siRNA] = 10. Viability of the cells was evaluated (% relative to the negative control) after 72 h of incubation with dendrimers and siRNAs. * indicates statistically significant differences (*p* < 0.05) from untreated cells.

Dendriplexes containing pro-apoptotic siRNAs (at 10-fold cation excess) have been shown to decrease the target cell viability, with the cytotoxic effect being the dose-response (Figure 8). The moderate cytotoxic effect of dendriplexes obtained at the siRNA concentration 100 nM suggest a potential use of these constructions in the combinational therapy, along with low-molecular chemodrugs. For instance, pro-apoptotic siRNAs carried by cationic dendrimers are efficiently combined, with 5-fluorouracil, as we have recently demonstrated [32]. These two agents act in synergy to induce cell death by affecting different mechanisms of the cell cycle regulation.

It is worth noting that, unlike the expected result, scramble siRNA appeared to exhibit some cytotoxic effect. Such an artifact is explained by the partial pairing of the scramble siRNA strands with mRNAs of the genes that are essential for normal cell functioning (5-methyltetrahydrofolate-homocysteine methyltransferase, kinesin family member 3B and DNA topoisomerase I), resulting in an inhibition of their translation [32]. These effects are not well-pronounced at relatively low siRNA concentrations; however, at 250 nM, the cytotoxic effect of scramble siRNA was as high as that of target siRNAs, suggesting a strong off-target effect limiting the use of the dendriplexes at high concentrations.

## 4. Conclusions

To use dendrimers as carriers for therapeutic nucleic acids, their compositions should be optimized to achieve better efficiency. Along with other factors, the dendrimer:NA ratio appears to have a considerable effect on the biological activities of the dendriplexes. Using cationic carbosilane dendrimers and anti-cancer siRNAs as a convenient model, we have summarized the data on the siRNA complexation obtained by several indirect methods, to find the optimal composition for dendriplexes for the siRNA delivery. Based on our findings, we have suggested a scheme of stepwise dendriplexes formation consisting the NA binding step, and further rearrangements of dendriplexes upon the increase of the dendrimer excess. The dendriplexes containing pro-apoptotic siRNAs Bcl-2, Bcl-xL, and Mcl-1 induced the cell death in both HeLa and HL-60 cell lines. This evidences that cationic carbosilane dendrimers are versatile agents to transfect both adherent and suspension cell lines, with the latter being known to be resistant to common synthetic vectors. The results obtained provide useful practical data for the design of dendrimer-based gene therapy tools.

## Figures and Tables

**Table 1:** Parameters of siRNA complexation by dendrimers BDEF32 (G2) and BDEF33 (G3), as calculated from the biophysical assays.

Assay	MR_50_ G2/G3	MR_sat_ G2/G3	CR_50_ G2/G3	CR_sat_ G2/G3
Circular dichroism	2.5/1	5/2.5	0.75/0.6	1.5/1.5
EB intercalation ^1^	3/1.5	5/3	0.9/0.9	1.5/1.8
Electrophoresis	3.3/1.3	6.7/3.3	1/0.75	2/2
Zeta potential ^2^	12/4	20/10	4/2.4	6/6

^1^ Points of global minima (Figure 2) were taken as MR_50_; ^2^ points of zero crossing (Figure 5) were taken as MR_50_.

## Supplementary Materials

The Supplementary Materials are available online at http://www.mdpi.com/1999-4923/11/1/25/s1. **Table S1**. Sequences of the oligonucleotides used. **Figure S1**. EBIA profiles for the dendriplexes. **Figure S2**. CD spectroscopy curves and ellipticity changing profiles for the dendriplexes. **Figure S3**. Zeta-potential profiles of the dendriplexes.
