# Supplementary material for: Complexes of Pro-Apoptotic siRNAs and Carbosilane Dendrimers: Formation and Effect on Cancer Cells
Source: Pharmaceutics. 2019 Jan 10;11(1):25. doi: 10.3390/pharmaceutics11010025 (PMC6359069; doi:10.3390/pharmaceutics11010025)
Supplement: Supplementary file 1 [file pharmaceutics-11-00025-s001.pdf]

pharmaceutics

Supplementary Materials:

Complexes of Pro-Apoptotic siRNAs and Carbosilane Dendrimers: Formation and Effect on Cancer Cells

Olga A. Krasheninina [1,†], Evgeny K. Apartsin [1,*,†], Elena Fuentes [2,3], Aleksandra Szulc [4], Maksim Ionov [4], Alya G. Venyaminova [1], Dzmitry Shcharbin [5], F. Javier de la Mata [2,3,6], Maria Bryszewska [4] and Rafael Gómez [2,3,6,*]

[1] Institute of Chemical Biology and Fundamental Medicine SB RAS, 630090 Novosibirsk, Russia; okrasheninina@gmail.com (O.A.K.); ven@niboch.nsc.ru (A.G.V.)
[2] Departamento de Química Orgánica y Química Inorgánica, UAH-IQAR, Universidad de Alcalá, 28805 Alcalá de Henares, Spain; elena.fuentes.paniagua@gmail.com (E.F.); javier.delamata@uah.es (F.J.M.)
[3] Networking Research Center on Bioengineering, Biomaterials and Nanomedicine (CIBER-BBN), Madrid, Spain
[4] Department of General Biophysics, University of Lodz, 90-236 Lodz, Poland; aleksandra_szulc@interia.pl (A.S.); maksion@biol.uni.lodz.pl (M.I.); marbrys@biol.uni.lodz.pl (M.B.)
[5] Institute of Biophysics and Cell Engineering of NASB, 220072 Minsk, Belarus; d.shcharbin@gmail.com (D.S.)
[6] Instituto Ramón y Cajal de Investigación Sanitaria, IRYCIS, Madrid, Spain
* Correspondence: eka@niboch.nsc.ru (E.K.A.); rafael.gomez@uah.es (R.G.); Tel.: +7-383-363-5129 (E.K.A.); +34-91-885-4685 (R.G.)
† These authors contributed equally to the work.

Table S1. Sequences of the oligonucleotides used.

Name	Sequence	Molecular weight	
		Calcd, $[M+H]^+$	Found,[1] *m/z*
**Bcl-2 S**	5'-r(GCUGCACCUGACGCCCUUC)dTdT-3'	6559.0	6559.5
**Bcl-2 AS**	5'-r(GAAGGGCGUCAGGUGCAGC)dTdT-3'	6805.2	6806.1
**Bcl-xL S**	5'-r(CAGGGACAGCAUAUCAGAG)dTdT-3'	6572.0	6572.4
**Bcl-xL AS**	5'-r(CUCUGAUAUGCUGUCCCUG)dTdT-3'	6712.2	6712.6
**Mcl-1 S**	5'-r(GGACUUUUAUACCUGUUAU)dTdT-3'	6757.2	6757.5
**Mcl-1 AS**	5'-r(AUAACAGGUAUAAAAGUCC)dTdG-3'	6562.0	6562.4
**Scr1**	5'-r(ACUCUAGCGGCACCAUCGUGCC)dTdT-3'	7562.7	7563.2
**Scr2**	5'-r(GGCACGAUGGUGCCGCUAGAGU)dTdT-3'	7722.8	7723.2

[1] MALDI TOF mass spectrometry.

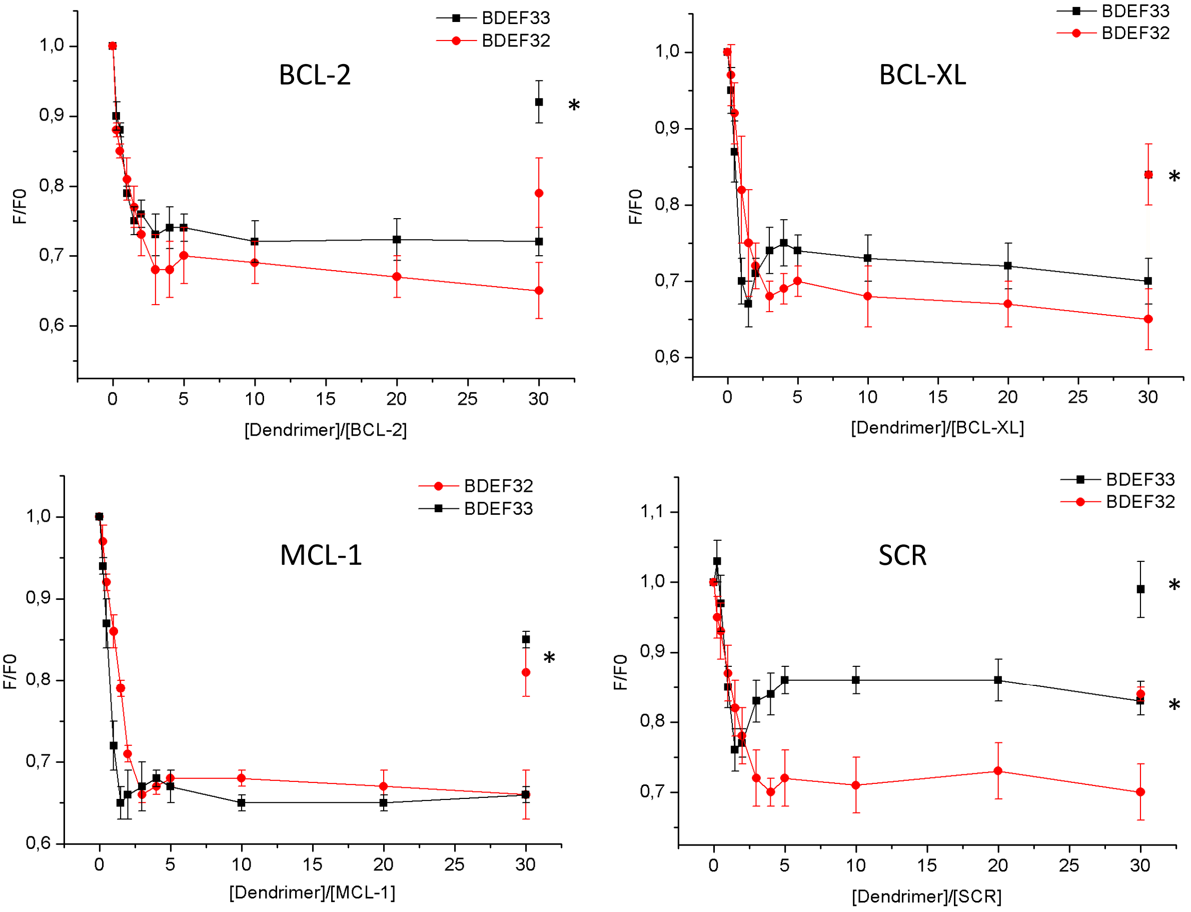

Figure S1. Changing of the normalized fluorescence intensity (at 595 nm) of ethidium bromide upon the titration of siRNA ([siRNA] = 0.3 μM, [EB] = 3 μM) by carbosilane dendrimers (* after heparin treatment). Conditions: 1×PBS (137 mM NaCl, 2.7 mM KCl, 10 mM phosphate buffer, pH 7.4), 25 °C.

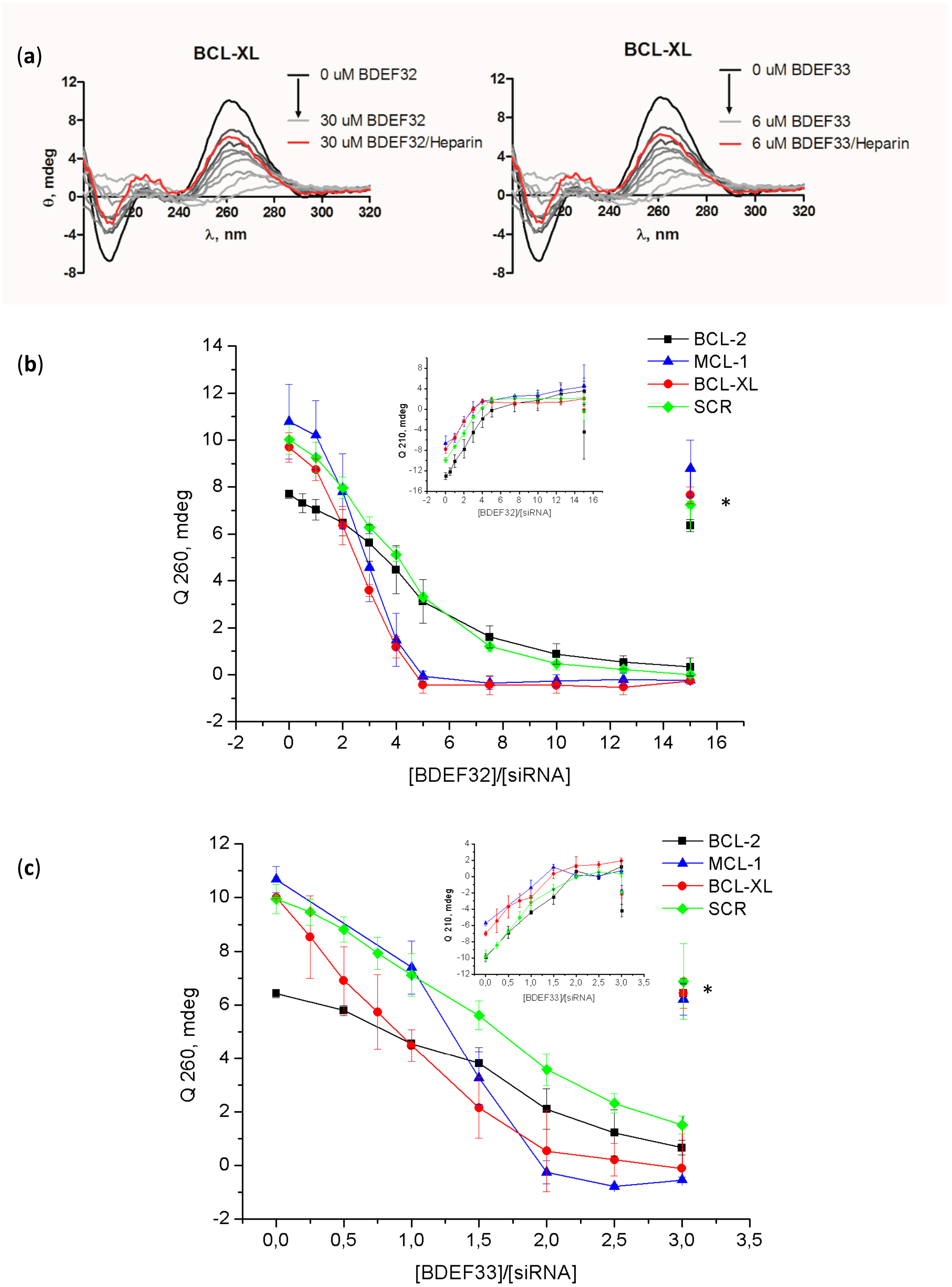

Figure S2. CD spectroscopy curves of siRNA Bcl-xL ([siRNA Bcl-xL] = 2 μM) in the presence of the carbosilane dendrimers of BDEF32 and BDEF33 (**a**); changes of ellipticity (at 260 nm) of dendriplexes formed by siRNAs and dendrimers BDEF32 (**b**) and BDEF33 (**c**) (* after heparin treatment). Conditions: 1×PBS (137 mM NaCl, 2.7 mM KCl, 10 mM phosphate buffer, pH 7.4), 25 °C.

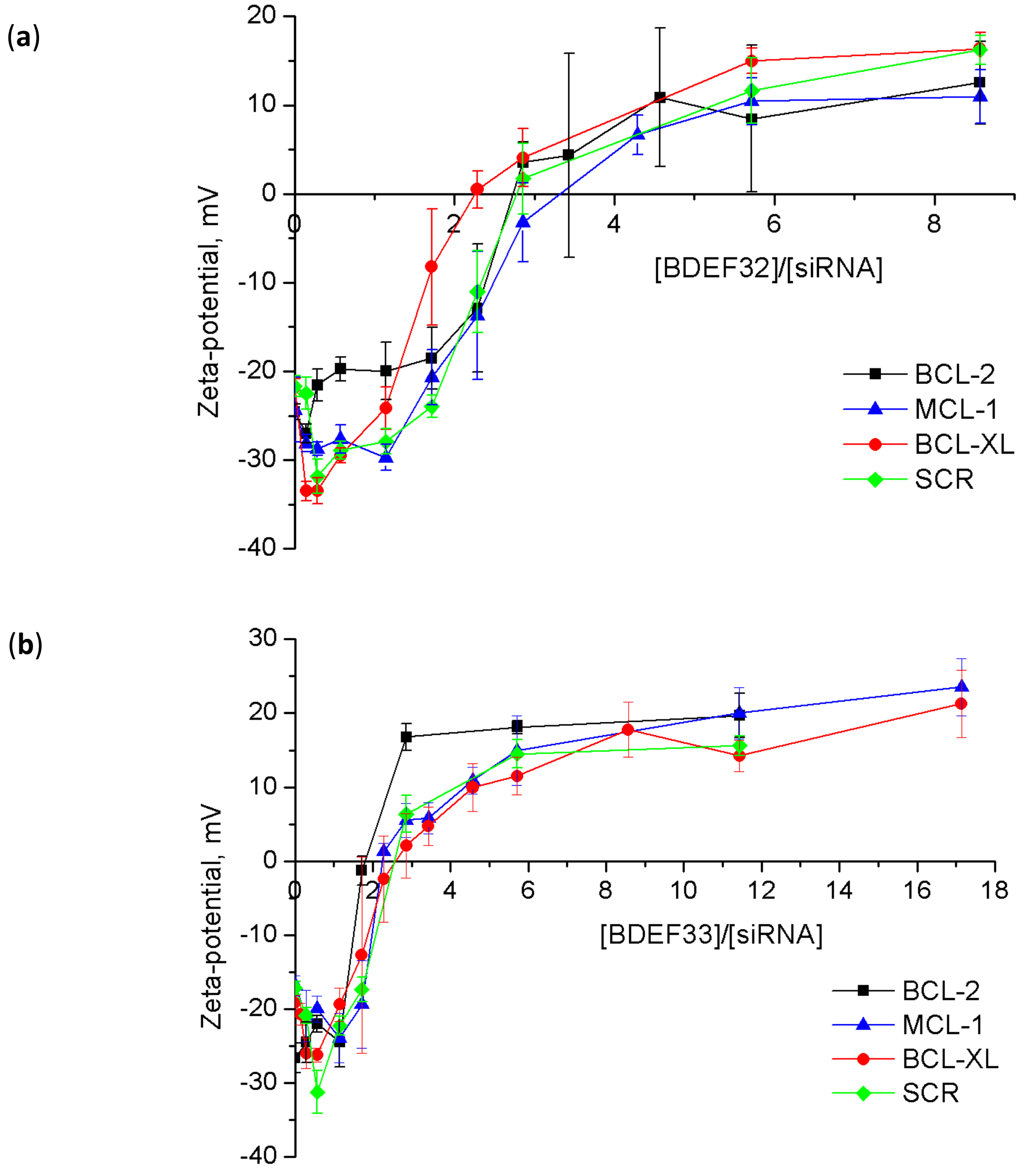

Figure S3. Zeta-potential profiles of the dendriplexes ([siRNA] = 0.5 μM) as a function of the concentration of carbosilane dendrimers of the second (**a**) and third (**b**) generations. Conditions: 1×PBS (137 mM NaCl, 2.7 mM KCl, 10 mM phosphate buffer, pH 7.4), 25 °C.
